# Exploring the effect of digital hoarding in the workplace on employee work performance

**DOI:** 10.3389/fpsyg.2025.1198825

**Published:** 2025-12-18

**Authors:** Changchun Gao, Chenhui Yu

**Affiliations:** Glorious Sun School of Business and Management, Donghua University, Shanghai, China

**Keywords:** digital hoarding in the workplace, thriving, job burnout, employee work performance, prevention focus, promotion focus

## Abstract

**Background:**

Academic viewpoints on the psychological impact of digital hoarding remain fragmented, and there is a lack of literature exploring the mechanism through which digital hoarding affects job performance in the workplace context.

**Method:**

This research draws on three quantitative studies—a primary study (*N* = 211) and two robustness checks (*N* = 114; *N* = 259)—to examine the effects of digital hoarding in the workplace on employee work performance. Data were analyzed by structural equation modeling, bootstrap procedures, and simple slope analysis.

**Conclusion:**

The findings show that digital hoarding in the workplace positively predicts job performance. Meanwhile, Job burnout exerts a negative mediating effect in this relationship, whereas thriving serves as a positive mediator. Besides, prevention focus significantly attenuates the positive association between digital hoarding and job performance, while the moderating role of promotion focus is not statistically significant.

**Discussion:**

These results contribute meaningfully to both theory and practice by advancing our understanding of how digital hoarding interacts with individual emotional states and work outcomes. Furthermore, they offer actionable insights for promoting employee wellbeing through health-oriented media use practices and for enhancing organizational effectiveness via performance-driven media management strategies.

## 1 Introduction

Digital social media platforms have been widely used among young people in China, enabling users to save massive contents in their favorites (Tandoc et al., 2019; Sillence et al., 2023). Under the tension of information explosion and digital attachment, data hoarding has emerged as a particularly prevalent information storage behavior in the process of using digital media. Digital hoarding refers to the behavior in which individuals retain electronic files that are not immediately necessary during digital media use and preserve them over an extended period (Neave et al., 2019; Sedera et al., 2022; Mu et al., 2025). From the perspective of media psychology, digital hoarding occupies an intermediate position on the continuum between hoarding disorder and minimalism (Cheng et al., 2025; Vital et al., 2018). In recent years, scholarly interest in the literature has shifted from the definition, measurement, and antecedents of digital hoarding to its effects on mental health and user behavior (Çelik, 2025; Liu et al., 2024; Wang et al., 2023). Is data hoarding a form of knowledge preservation or a source of psychological stress? Researchers have continued to debate the role of digital hoarding up to now. At present, empirical evidence on this issue is still scarce, so it is necessary to further explore the impact of digital hoarding on mental health and behavioral performance.

Current research primarily investigates the negative effects of digital hoarding on mental health from perspectives of psychoanalytic and behaviorism (Sweeten et al., 2018; Thorpe et al., 2019). Within psychoanalytic, Fromm’s theory of the hoarding personality suggests that digital hoarding reflects a coping mechanism among individuals with anxious attachment, who accumulate digital items to alleviate distress and enhance psychological security. From a psychoanalytic standpoint, digital hoarding represents an extension of material hoarding into virtual space, reinforcing patterns of possessive behavior, diminishing subjective experiences of selfhood, and exacerbating anxiety (Sweeten et al., 2018). Grounded in the SOR framework, behaviorism examines the mechanisms through which digital media characteristics—such as digital content, storage capacity, and functional interface design—influence individual tendencies toward digital hoarding and emotional exhaustion (Thorpe et al., 2019). Under conditions of information overload, such hoarding behaviors may impose excessive cognitive demands on individuals, potentially leading to mental fatigue and burnout (Liu and Liu, 2025). Based on psychoanalysis and behaviorism, a series of empirical studies have uncovered the dark side of digital hoarding, including anxiety, hoarding disorder, and worry (Luxon et al., 2019; Zaremohzzabieh et al., 2024; Koklic et al., 2025).

A growing body of literature employs a positive psychology perspective to examine the beneficial impacts of digital hoarding on individual mental health. Grounded in conservation of resources theory, this perspective posits that the online materials accumulated through digital hoarding behaviors serve valuable functions—such as preserving evidence, preparing for potential future needs, and supporting self-directed learning—which contribute to the expansion of personal resource reservoirs and may enhance individuals’ self-efficacy and psychological resilience (McKellar et al., 2020). Mehrotra et al. (2025) conducted a qualitative study grounded in the TCV-SECI framework, revealing that individuals engage in knowledge creation through the process of digital hoarding. Their findings suggest that digital content accumulation does not inherently result in information overload; rather, it may facilitate structured knowledge organization and encourage knowledge-sharing behaviors, thereby supporting individual learning. Jia et al. (2025) conducted a study on researchers in the humanities, revealing a positive correlation between digital hoarding and the perceived affordances of digital content, with beneficial effects on individual efficacy and academic achievement. The aforementioned literature based on psychoanalysis and behaviorism, taking daily entertainment and consumption scenarios as the entry point, generally supports the negative impact of digital hoarding (Luxon et al., 2019; Zaremohzzabieh et al., 2024; Koklic et al., 2025). In contrast, the literature based on positive psychology, taking the workplace as the entry point, has discovered fragmented evidence that digital hoarding has a positive effect on employees’ psychology and performance (McKellar et al., 2020; Mehrotra et al., 2025; Jia et al., 2025).

A critical review of the existing literature reveals three significant research gaps. First, empirical studies have not to reach a consensus on the psychological implications of digital hoarding (Zaremohzzabieh et al., 2024; Koklic et al., 2025; Mehrotra et al., 2025). Given that research in this domain is still in its early stages and the accumulation of empirical evidence remains limited, the effects of digital hoarding on mental health and behavioral outcomes remain ambiguous and insufficiently understood. Second, while current scholarship predominantly examines digital hoarding within the context of everyday personal media use, there is a notable paucity of research investigating its impact in organizational settings—particularly among enterprise employees—and its potential consequences for work performance (Wu et al., 2024). Most studies rely on self-reported usage of consumer-oriented platforms such as TikTok, Facebook, and X when administering surveys, with minimal attention paid to digital hoarding behaviors involving workplace-specific tools, including enterprise communication systems, SaaS applications, and personal cloud storage used in professional contexts. Third, although emerging studies have begun to identify boundary conditions that may moderate the relationship between digital hoarding and individual outcomes, this body of knowledge remains fragmented and underdeveloped. For example, Liu and Liu (2025), as well as Koklic et al. (2025), have examined the moderating roles of trait mindfulness and organizational culture, respectively. However, such investigations are still rare and call for more systematic exploration.

To address the aforementioned research gaps, this study investigates the underlying mechanisms through which digital hoarding affects employee work performance by drawing on cognitive load theory, resource conservation theory, and regulatory focus theory. Three empirical studies were conducted to test the proposed hypotheses. In the primary study, data were collected via a questionnaire survey administered to 211 employees across six enterprises from 2021 to 2022. SEM, bootstrap method and simple slope plot were employed to examine the effects of workplace digital hoarding on job burnout, thriving, and job performance, as well as the moderating roles of defensive and promotion-focused regulatory orientations. In the first robustness check, recognizing that measurement variations in digital hoarding may influence results, an alternative scale was adopted to assess digital hoarding behavior, and data from 114 employees in two internet companies were collected to further validate the main effect of workplace digital hoarding on job performance in 2023. In the second robustness test, dataset was gathered from 259 employees across seven organizations in 2024. This study further examined the robustness of the parallel mediating effects of job burnout and thriving between digital hoarding at workplace and work performance. These studies reveal the double-edged sword effect of digital hoarding on employees’ work performance and provide corresponding practical implications for enterprises’ digital media management.

## 2 Theoretical framework and hypothesis development

### 2.1 The impact of workplace digital hoarding on employees’ work performance

Work performance is a multidimensional concept that generally includes task performance, relationship performance, learning performance, and innovation performance. From the perspective of COR, empirical evidence consistently supports the positive influence of various resource categories—such as material capital, human capital, social capital and psychological capital—on work performance. Given that digital hoarding enables the accumulation of valuable digital resources that can function as instrumental assets in work processes, it is plausible that such accumulated online resources may serve as a supplementary resource reservoir, thereby enhancing employees’ capacity to perform effectively.

Digital hoarding in the workplace refers to the behavior of employees continuously increasing content in digital office media and being reluctant to delete it. McKellar et al. (2020) were the first to observe the causes of digital hoarding among employees in the workplace. Guided by the Theory of Planned Behavior, they found that alleviating anxiety, responsibility avoidance, behavioral compliance, and information collection are the main motivations for employees’ digital hoarding. Therefore, under the assumption of rational decision-making, the hoarded content itself has functional value, enriching employees’ work resources and enhancing their psychological ownership. Mehrotra et al. (2025) also supported the view that digital hoarding promotes knowledge transformation and thereby improves innovation performance through qualitative research. Currently, there is a lack of quantitative research providing statistical evidence on the relationship between digital hoarding in the workplace and work performance. McKellar et al. (2024) used a questionnaire method with a small sample of 11 employees to support the positive effect of digital hoarding on work performance. Tugtekin and Tugtekin (2025) took 213 scholars in the academic field as samples and found through the PLS-SEM method that digital hoarding has a positive impact on research performance.

*H1:* Digital hoarding in the workplace has a positive impact on employees’ work performance.

### 2.2 The mediating effect of job burnout

Job burnout is a syndrome characterized by emotional exhaustion, depersonalization and reduced personal accomplishment (Maslach, 2012). Specifically, burnout is a persistent, negative, work-related psychological state that occurs in normal people (Maslach, 2012). From the perspectives of psychoanalysis and behaviorism, negative psychological factors such as anxiety, depression, and hoarding personality disorder may lead to burnout.

Grounded in cognitive load theory, excessive information and environmental cues can substantially deplete an individual’s attentional resources, leading to mental fatigue and cognitive dysfunction (Head and Helton, 2014). This prolonged state of cognitive strain constitutes a potent negative experience that may directly contribute to core dimensions of job burnout—specifically emotional exhaustion and depersonalization. Supporting this pathway, Liu and Liu (2025) conducted a study with 801 participants and confirmed a significant positive relationship between digital hoarding and both subjective fatigue and cognitive impairment. Further evidence comes from Mu et al. (2025), who, in assessing the predictive validity of digital hoarding, identified positive associations with clinical constructs such as hoarding disorder, anxiety, and obsessive-compulsive tendencies—all of which are established antecedents of burnout. Additionally, Agarwal et al. (2024) and Koklic et al. (2025) independently employed survey-based methodologies to investigate consumer behaviors related to digital product accumulation. Their findings consistently indicate that digital hoarding is positively associated with heightened levels of worry and anxiety, while also negatively impacting subjective wellbeing. Collectively, these results suggest that digital hoarding may exacerbate psychological strain, thereby increasing vulnerability to burnout.

*H2:* Digital hoarding in the workplace has a positive impact on employee burnout.

COR posits that emotional resources serve as a fundamental prerequisite for sustaining work motivation. When individuals experience depletion or scarcity of such resources, they are more likely to withdraw cognitively, emotionally, and behaviorally from their work roles—a process reflected in reduced work engagement—which subsequently undermines their job performance. Demerouti et al. (2005) provided empirical support for the negative impact of burnout on employee job performance, establishing a foundational link within the job demands-resources framework. Prentice and Thaichon (2019), focusing on hotel employees, re-examined and confirmed this relationship, further substantiating the detrimental role of burnout in undermining work performance in service-oriented settings. More recently, Miao et al. (2025) employed panel data to validate the negative relationship between burnout and job performance, offering robust evidence through advanced statistical modeling. Collectively, these studies consistently demonstrate that elevated levels of burnout are associated with diminished job performance across diverse occupational contexts.

*H3:* Burnout has a negative impact on employees’ work performance.

*H4:* Burnout plays a mediating role in the relationship between digital hoarding and employees’ work performance.

### 2.3 The mediating effect of thriving at work

Thriving represents the integration of hedonic wellbeing and eudaimonic wellbeing, encompassing both the dimensions of learning and personal growth (Waterman, 2008; Ryan and Deci, 2001). Specifically, thriving at work includes multiple aspects such as satisfaction, positive emotions, a sense of support, a sense of belonging, participation, skills, and learning (Su et al., 2014).

According to COR, digital hoarding at workplace enhances individuals’ access to information necessary for coping with uncertainty and provides learning resources for employees, thereby contributing to the development of psychological capital—including self-efficacy, optimism, and resilience (McKellar et al., 2024). Thriving is conceptualized as comprising two core dimensions: learning and vigor. Empirical evidence indicates that entertainment-driven digital hoarding positively enhances individuals’ passion and vigor, whereas learning-oriented digital hoarding supplies knowledge resources that support employee learning and skill development (McKellar et al., 2020; Mehrotra et al., 2025).

*H5:* Digital hoarding in the workplace has a positive effect on thriving at work.

Work performance comprises four key dimensions: task performance, relationship performance, innovation performance, and learning performance. According to COR, thriving—understood as a positive emotional resource—can enhance employees’ work engagement and learning motivation, thereby positively influencing both learning and task performance (Chang et al., 2024; Barnes and Collier, 2013). Empirical evidence shows that vitality contributes to the maintenance of individuals’ physical and mental wellbeing and supports enhanced creativity, thus promoting innovation performance (Duan et al., 2018; Duan et al., 2016). Furthermore, thriving is positively associated with employees’ prosocial behaviors, which in turn facilitates improvements in relationship performance (Tarsuslu, 2024; Hart, 2024).

*H6:* Thriving at work has a positive effect on employees’ work performance.

*H7:* Thriving at work plays a positive mediating role in relationship between digital hoarding and employees’ job performance.

### 2.4 The moderating effect of regulatory focus

Regulatory focus theory, introduced by Higgins (1997), posits that individuals adopt two distinct regulatory modes when pursuing goals: promotion focus and prevention focus. These modes shape goal-directed behaviors by influencing motivational orientations and decision-making tendencies. Promotion focus is oriented toward the pursuit of gains and personal growth, leading individuals to emphasize the attainment of ideal outcomes. Such individuals are more likely to engage in proactive exploration, innovation, and risk-taking (Karagöz and Ramkissoon, 2024). In contrast, prevention focus is oriented toward avoiding losses and maintaining safety, with individuals prioritizing duty fulfillment and risk avoidance. Their behavior tends to be cautious, conservative, and rule-bound (Maduku, 2024).

Vinoi et al. (2024) examined the motivational underpinnings of digital hoarding from the perspective of two-factor theory, identifying two distinct types: defensive and promotive motivations. Their findings indicate that digitally hoarding driven by defensive motivations is closely associated with negative emotional experiences and adverse behavioral outcomes, whereas hoarding motivated by promotive goals contributes positively to individual behavioral performance. Empirical evidence demonstrates that when individuals retain digital content due to defensive motives—such as “just in case” or “for evidence”—their digital hoarding behavior positively predicts symptoms of obsessive-compulsive tendencies, anxiety, and insecure attachment (Luxon et al., 2019; Zaremohzzabieh et al., 2024; Koklic et al., 2025). In contrast, qualitative insights reveal that when digital hoarding stems from promotive motivations like information gathering and self-improvement, it is positively linked to perceived efficacy, personal growth, and enhanced performance (McKellar et al., 2020; Mehrotra et al., 2025; Jia et al., 2025).

*H8:* Prevention focus negatively moderates the relationship between digital hoarding at workplace and employees’ work performance.

*H9:* Promotion focus positively moderates the relationship between digital hoarding at workplace and employees’ work performance.

## 3 Research design

### 3.1 Data

The empirical analysis comprises three studies. The first is a primary study designed to test the proposed hypotheses, while the remaining two serve as robustness checks. The second study evaluates whether the main effect of workplace digital hoarding remains consistent when measured using alternative scales. The third study assesses the stability of the original main and mediating effects by employing data from different years.

Primary Study: During the period from March 2021 to December 2022, the team conducted a questionnaire survey of six companies located in Hangzhou, Nanjing and Shanghai. These six companies are all medium-sized firms in the IT industry, with an average of 127 employees. They were selected because IT enterprises typically engage in extensive digital workflows, leading to frequent instances of workplace digital hoarding among employees. Furthermore, given that access to digital resources varies across job roles, the resulting sample data exhibits sufficient variability, which is essential for empirical analysis. Among the participating organizations, two had been in operation for less than 3 years, three for 3–8 years, and one for more than 8 years. To mitigate single source bias, the questionnaire survey was divided into three processes. Process 1: the researcher collected data from enterprise employees on basic information and digital hoarding. Process 2: the researcher collected data from enterprise employees on thriving at work and job burnout. Process 3: the researcher investigated the employees’ work performance data. After matching the questionnaire data, the researcher distributed 60 questionnaires to each company, collected 287 questionnaires, and eliminated 76 invalid questionnaires. The questionnaire return rate and effective return rate were 79.72% or 58.61%, respectively, which basically met the criteria needed for the study.

The distribution of samples is shown as follows: first, 98 employees of 20–30 years old (Age = 1), 75 of 31–40 years old (Age = 2), and 38 of 41–50 years old (Age = 3); second, 100 male employees (Female = 0) and 111 female employees (Female = 1); third, 104 employees at the basic level (Power = 1), 83 at the middle management level (Power = 2), and 24 at the top management level (Power = 3); fourth, 26 employees with high school or secondary school education (Education = 1), 120 with undergraduate or college education (Education = 2), and 65 with graduate education or higher education (Education = 3).

Robustness test: (1) The first robustness test was conducted between November and December 2023, during which the research team administered questionnaires to employees at two Internet companies in Beijing, China. Both firms employed more than 150 individuals and had been in operation for over 5 years. A total of 120 questionnaires were distributed, of which 114 were returned and deemed valid for analysis. Given that this study focuses solely on testing the robustness of the main effect hypotheses, and considering the limited number of variables involved, the risk of severe common method bias is relatively low. Consequently, all data were collected simultaneously rather than in separate waves. (2) To further establish the robustness of the main effects and the mediating effects over time, data were collected from seven enterprises in IT industry across five cities—Qingdao, Jinan, Tianjin, Beijing, and Shijiazhuang—from March to July 2024. Among the sampled organizations, one had been in operation for less than 3 years, four for 3–8 years, and two for more than 8 years. A total of 350 questionnaires were distributed, with 50 administered per organization, yielding 259 valid responses.

### 3.2 Measurement

#### 3.2.1 Work performance

The explained variable is work performance (WP). According to Williams and Anderson (1991), Podsakoff et al. (1997) and Van der Vegt and Janssen (2003), four dimensions (task performance, relationship performance, learning performance and innovation performance) are extracted to measure Chinese employees’ work performance with 39 questions in questionnaire.

#### 3.2.2 Digital hoarding in the workplace

The explanatory variable is digital hoarding in the workplace (DHW). To rule out the possibility that inconsistent findings might arise from scale differences, distinct measurement scales were employed in the main study and the robustness tests to assess workplace digital hoarding.

In primary study, the researcher modified the original scale DBWQ based on Neave et al. (2019), which included the following six options: (1) I will try to expand the storage space of the device or application to increase the number of files; (2) I will try to use a variety of media management methods to increase the number of files; (3) I will add content that I am interested in or find useful to the device or application; (4) I will keep some videos, articles, or records for a long time because they are interesting; (5) I will keep some videos, articles, or records because of their potential use; and (6) I will not delete files from the device even after a long time has passed.

In both robustness tests, we adopted the DHQ scale developed by Sillence et al. (2023), which comprises two dimensions: content accumulation and deletion difficulty. The scale was adapted based on feedback from the preliminary survey, with each dimension measured using three items.

#### 3.2.3 Thriving at work and job burnout

The first mediating variable is thriving at work (TV). The scale developed by Porath et al. (2012) was used in this study to measure thriving at work, which consisted of 24 question items.

The second mediating variable is job burnout (JB). We referred to the scale of Maslach (2012) to measure job burnout, which consists of 15 items.

#### 3.2.4 Prevention focus and promotion focus

Regulation focus consists of prevention focus and promotion focus. We adopted the scale developed by Higgins (1997) to measure prevention focus and promotion focus, with each construct assessed using a 9-item subscale.

#### 3.2.5 Control variables

The control variables are age (Age), gender (Female), authority (Power), and education level (Education). The specific measurement ranges of these variables are described in the previous section.

### 3.3 Statistical methods

In the main study, structural equation modeling (SEM), bootstrap procedures, and simple slope plots were employed to test all research hypotheses. For the first robustness test, linear regression analysis was conducted to examine the main effect of digital hoarding in the workplace on work performance. In the second robustness test, bootstrap methods were applied to assess the parallel mediating roles of job burnout and thriving at work. We estimated the path coefficients of the SEM through MPlus5.0 to test the main effects and mediating effects. We used SPSS27.0 to examine the reliability and validity of each variable, the descriptive statistics, the moderating effect of PRE-F and PRO-F, and the parallel mediating effect based on the bootstrap procedures.

### 3.4 Descriptive statistics

Running SPSS27.0, we conducted descriptive statistics and correlation analysis on the sample data, and obtained the results shown in Table 1. From the perspective of descriptive statistics, there is some variability between the explanatory and dependent variables, making regression analysis appropriate. In addition, there is a significant correlation between independent variables, mediating variables, and dependent variables, which preliminarily validates the research hypothesis.

**Table 1:** Descriptive statistics.

Variable	Mean	SD	WP	DHW	TV	JB	PRE-F	PRO-F
WP	3.9617	0.9982	–	–	–	–	–	–
DHW	3.7747	1.1892	0.430**	–	–	–	–	–
TV	3.8804	1.1302	0.322**	0.196**	–	–	–	–
JB	3.8688	1.2680	−0.388**	0.411**	0.163**	–	–	–
PRE-F	3.6256	1.2921	−0.215*	0.127	−0.158	0.133	–	–
PRO-F	3.7845	0.9265	0.356**	0.058	0.114	−0.125	−0.619***	–

Statistical standard errors are in parentheses. *** indicates significant at the 0.01 level, ** indicates significant at the 0.05 level, and * indicates significant at the 0.1 level.

## 4 Empirical result

We present the empirical findings of the main study followed by two robustness tests. The main study analyzed data collected in 2022 (*N* = 211) by SEM, Bootstrap procedures and simple slope plots to test all proposed hypotheses (H1–H9). Robustness Test 1 employed linear regression analysis on an independent dataset from 2023 (*N* = 114) to re-examine the main effect hypothesis (H1). Given the existence of two distinct measurement approaches for DHW, this test was conducted to rule out potential biases arising from measurement instrument differences. Robustness Test 2 applied Bootstrap methods to a 2024 dataset (*N* = 259) to re-examine the mediation hypotheses (H2–H9). In addition to employing alternative measurement specifications, this test aimed to confirm the temporal stability of the dual-edged effect of DHW across different time points.

### 4.1 Reliability test and single source bias test

Because of the excessive number of question items for some of the variables, instead of showing the loading factors for each question item, this study provides the Cronbach’s *α*, AVE, and CR coefficients for each variable. The results of the reliability tests are as follows: (1) Cronbach’s alpha coefficient for job performance is 0.9046, AVE value is 0.7750, and CR value is 0.8732; (2) Cronbach’s alpha coefficient for digital hoarding is 0.9317, AVE value is 0.7775, and CR value is 0.9129; (3) Cronbach’s alpha coefficient for thriving at work is 0.8815, AVE value is 0.7376, and CR value is 0.9335; (4) Cronbach’s alpha coefficient for job burnout is 0.9258, AVE value is 0.7447, and CR value is 0.9357. (5) Cronbach’s alpha coefficient for prevention focus is 0.8924, AVE value is 0.6266, and CR value is 0.8711. (6) Cronbach’s alpha coefficient for promotion focus is 0.8529, AVE value is 0.7935, and CR value is 0.8992. The Cronbach’s alpha coefficients of all variables were greater than 0.85, which indicates that the measurement results of the questionnaire are relatively reliable. The AVE values of all variables were greater than 0.6and CR values were greater than 0.8, which indicates that the validity of the questionnaire is high and the questionnaire suitable for factor analysis.

Although this study was divided into multiple questionnaire distributions to mitigate single source bias, it may still exist. For this reason, this study used Harman’s one-factor test to measure single source bias, and factor analysis was conducted on all variable data processes. The cumulative contribution of the first component factor reached 27.14%, which was below the 40% threshold, so the questionnaire data did not have serious single source bias.

### 4.2 The empirical results of main study

**Figure 1:**
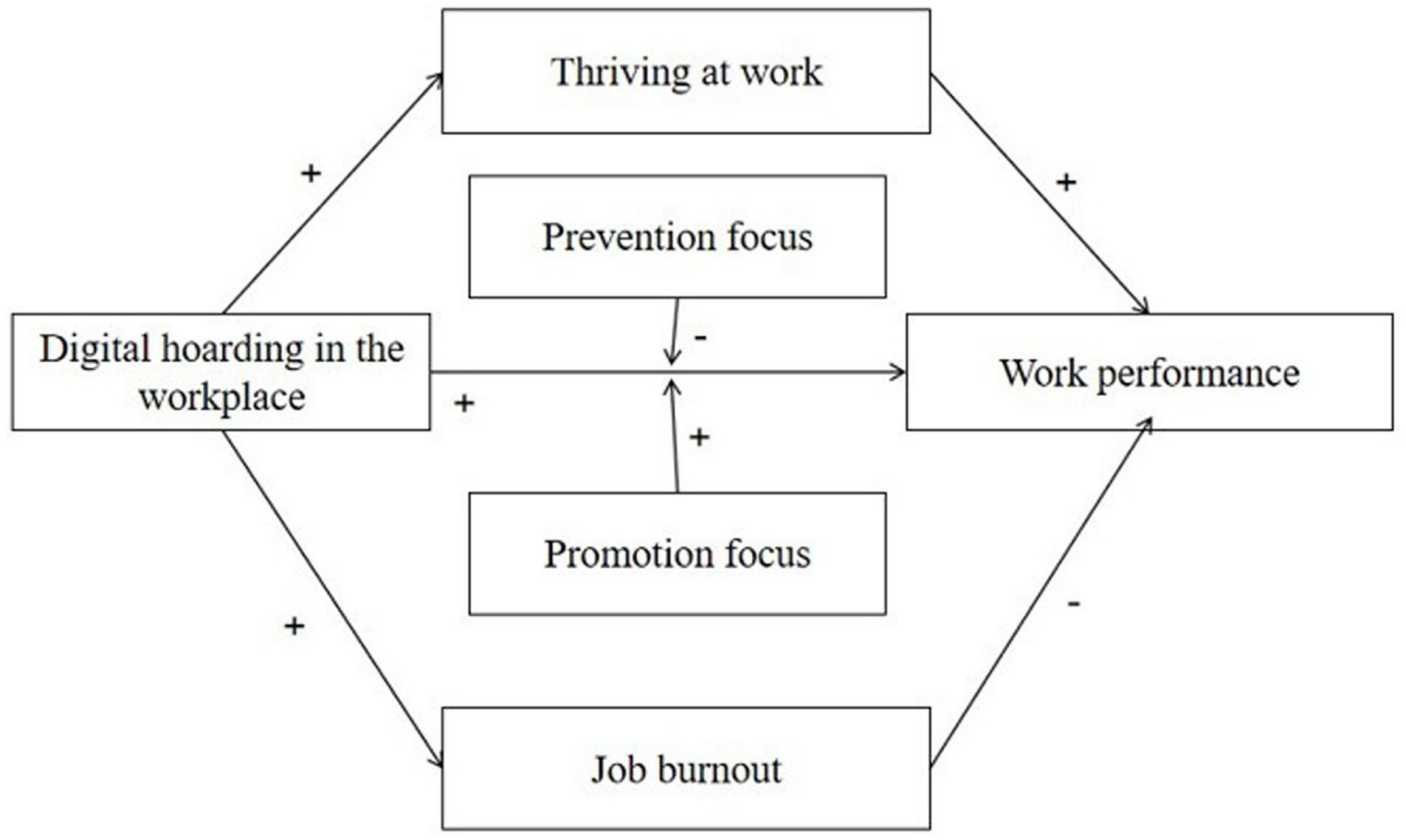
Theoretical model.

The main study tests all the hypotheses—from H1 to H9. By running Mplus 5.0, we used SEM to test the main effects and mediating effects, and obtained the statistical results as shown in Table 2. The non-standardized and standardized path coefficients were consistent across all paths and significant at the 0.01 level. First, the standardized coefficient of DHW on WP was 0.397 (SE = 0.069, *p* < 0.01), supporting the direct effect of workplace digital hoarding. Second, DHW was positively related to JB (*β* = 0.306, SE = 0.053, *p* < 0.01), and JB was negatively associated with WP (*β* = −0.229, SE = 0.084, *p* < 0.01), indicating that JB mediates the relationship between DHW and WP. Third, DHW was positively linked to TV (*β* = 0.480, SE = 0.063, *p* < 0.01), and TV was positively related to WP (*β* = 0.207, SE = 0.080, *p* < 0.01), supporting the mediating role of TV in the DHW–WP relationship. Collectively, these findings provide initial support for hypotheses H1–H7 (Figure 1 and Table 3).

**Table 2:** Regression results.

Path	Non-standardized coefficient	Standardized coefficient	SE	*Z*	*p*
DHW → WP	0.354	0.397	0.069	5.096	0.000***
TV → WP	0.223	0.207	0.08	2.805	0.005***
JB → WP	−0.287	−0.229	0.084	−3.414	0.001***
DHW → TV	0.396	0.48	0.063	6.318	0.000***
DHW → JB	0.217	0.306	0.053	4.076	0.000***
Main study: *N* = 211					

Statistical standard errors are in parentheses. *** indicates significant at the 0.01 level, ** indicates significant at the 0.05 level, and * indicates significant at the 0.1 level.

**Table 3:** The goodness of fit of structural equation models.

Index	*χ* ^2^	df	*p*	*χ*^2^/df	GFI	RMSEA	RMR	CFI	NFI	NNFI
Reference value	–	–	>0.05	<3	>0.9	<0.10	<0.05	>0.9	>0.9	>0.9
Actual value	607.777	555	0.060	1.095	0.927	0.021	0.039	0.988	0.877	0.987

Meanwhile, most fit indices of the SEM met conventional criteria for model fit, indicating that the model exhibits adequate goodness of fit. NFI = 0.877, which did not reach the threshold value of 0.9, but is relatively close to it. Therefore, the SEM generally has good explanatory power.

Given the relatively small sample size and the potential for left-skewed distribution, we further employed the bootstrap procedure to test the parallel mediating effects of JB and TV under the condition of 5,000 repeated samplings. Table 4 presents the calculation results of the indirect effect. In the parallel mediation model, the value of the indirect effect (DHW → TV → WP) is 0.081 (SE = 2.532, *p* < 0.05), while the value of the indirect effect (DHW → JB → WP) is −0.054 (SE = 0.023, *p* < 0.05). It further supports the conclusions of H2-H7.

**Table 4:** The result of the Bootstrap procedure.

Path	Indirect effect	Boot SE	*Z*	*p*	95%BootCI
DHW → TV → WP	0.081	0.032	2.532	0.012**	0.031–0.158
DHW → JB → WP	−0.054	0.023	2.392	0.018**	−0.108 to −0.018
Main study: *N* = 211					

Statistical standard errors are in parentheses. *** indicates significant at the 0.01 level, ** indicates significant at the 0.05 level, and * indicates significant at the 0.1 level.

**Figure 2:**
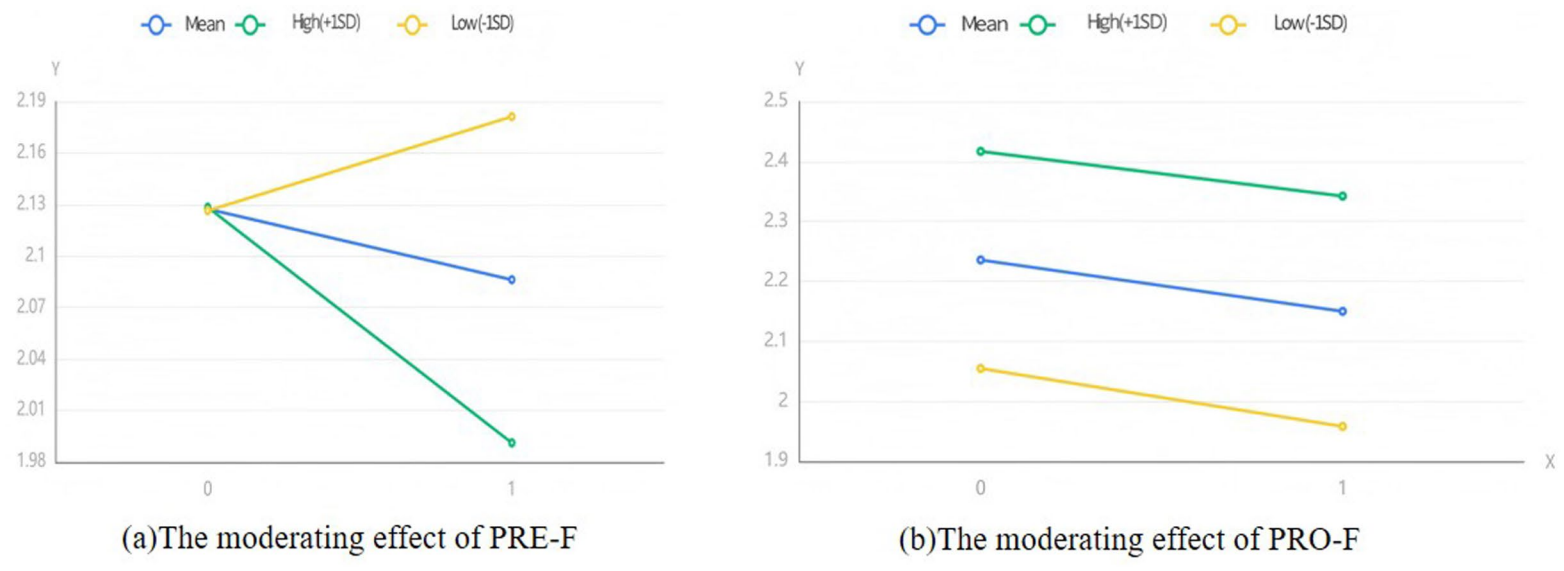
Results of the moderating effect.

Running SPSS 27.0, the regression equation included four control variables: Age, Female, Power, and Education, and examined the moderating effects of PRE-F and PRO-F. As shown in Figure 2, we present the moderating effects of PRE-F and PRO-F in the form of simple slope graphs. The results showed that the coefficient of the interaction term between PRE-F and DHW was −0.1 (SE = 0.055, *p* < 0.10), which was significant at the 0.1 level. This result indicates that H8 can be accepted. However, the coefficient of the interaction term between PRO-F and DHW was −0.013 (SE = 0.07, *p* > 0.10), which was not significant. This result was inconsistent with the hypothesis, and thus H9 was rejected (Tables 5, 6).

**Table 5:** The result of robustness test 1.

Variable	Beta	S.E.	*t*	*p*	VIF	*R* ^2^	Adj-*R*^2^	*F*
Constant	2.054	0.297	6.917	0.000***	–	0.429	0.422	*F* = 33.3, *p* = 0.000***
DHW	0.461	0.08	5.771	0.000***	1			
Control	Yes							
Robustness test 1: *N* = 114								

Statistical standard errors are in parentheses. *** indicates significant at the 0.01 level, ** indicates significant at the 0.05 level, and * indicates significant at the 0.1 level.

**Table 6:** The result of robustness test 2.

Path	Indirect effect	Boot SE	*Z*	*p*	95%BootCI
SSR → JB → WF	−0.025	0.012	2.083	0.043**	−0.075 to −0.011
SSR → IN→WF	0.129	0.036	3.609	0.000***	0.071–0.211
Robustness test 2: *N* = 259					

Statistical standard errors are in parentheses. *** indicates significant at the 0.01 level, ** indicates significant at the 0.05 level, and * indicates significant at the 0.1 level.

### 4.3 The empirical results of robustness test 1

The first robustness test focuses specifically on the main effect hypothesis (H1). Different scholars have used different scales to measure DHW, so we need to ensure that the conclusion is not affected by the measurement method. In view of this, our first robustness test re-collected data from 114 employees using another scale. Under the premise of controlling variables, this study only examined the main effect of workplace digital hoarding through a linear regression model. The results showed that the F-statistic of the linear regression was 33.3 (*p* < 0.01), the adj-R square was 0.422, the model was overall significant, and the explanatory power was good. In addition, the regression coefficient of DHW on WP was 0.461 (SE = 0.08, *p* < 0.01), further supporting the reliability of H1.

### 4.4 The empirical results of robustness test 2

The second robustness test further evaluates the parallel mediating effects (H2–H9). In the second robustness test, to ensure the stability and generalizability of the statistical conclusions over time, we employed the Bootstrap procedure with 5,000 resampling iterations to analyze the data from a sample of 259 employees. Run SPSS27.0, the value of the indirect effect (DHW → JB → WP) was −0.025 (SE = 0.012, *p* < 0.05), indicating that JB played a negative mediating role between DHW and WP. Additionally, the value of the indirect effect (DHW → TV → WP) was 0.129 (SE = 0.036, *p* < 0.01), suggesting that TV played a positive mediating role between DHW and WP. Although the coefficients and significance levels have slightly changed, H2-H7 were once again verified.

## 5 Discussion

### 5.1 The impact of digital hoarding in the workplace on job performance

This research indicates that digital hoarding in the workplace generally has a positive impact on employees’ work performance. Since Van Bennekom et al. (2015) identified a link between digital hoarding and obsessive-compulsive tendencies, scholarly debate has persisted over its implications for individuals’ psychological wellbeing and behavioral outcomes. While numerous quantitative studies have documented the detrimental effects of digital hoarding, evidence supporting its beneficial role—often derived from qualitative research—remains scattered and fragmented. Situating our inquiry in the workplace context, this study empirically confirms the positive impact of digital hoarding on employee work performance through multiple survey-based analyses. By providing robust quantitative support, this research strengthens the argument that digital hoarding can serve as a performance-enhancing factor and aligns with the findings of Jia et al. (2025).

COR posits that work resources enhance employees’ capacity to cope with challenges, thereby reducing work-related stress and fostering greater work engagement. From a COR perspective, digital hoarding enables employees to accumulate valuable informational and cognitive resources in the workplace—resources that may not be immediately utilized but retain latent potential to support future task performance (Thorpe et al., 2019). Therefore, viewing digital hoarding solely as a maladaptive behavior is overly simplistic. Instead, it is essential to acknowledge both the resource-acquisition function of digital hoarding and its positive implications for work performance.

### 5.2 The parallel mediating effect of job burnout and thriving

Our study reveals that job burnout exerts a negative mediating effect between workplace digital hoarding and job performance, whereas vigor functions as a positive mediator. Prior research has typically addressed the question of whether digital hoarding accumulates resources or anxiety through single-path mechanisms, often emphasizing one perspective at the expense of the other (Luxon et al., 2019; Zaremohzzabieh et al., 2024; McKellar et al., 2020; Mehrotra et al., 2025). In contrast, this study adopts an integrative framework grounded in cognitive behaviorism and positive psychology, simultaneously incorporating both job burnout and vigor into the theoretical model to examine the dual nature—or double-edged sword effect—of digital hoarding on work performance. The empirical findings successfully reconcile divergent scholarly perspectives by demonstrating that both pathways coexist, thereby helping to bridge the theoretical divide concerning the consequences of digital hoarding.

Grounded in the cognitive behaviorism framework, we draw upon attention overload theory to argue that the excessive information accumulated through digital hoarding may impose a heavy cognitive load on employees, leading to cognitive fatigue (Koklic et al., 2025; Liu and Liu, 2025). This fatigue, in turn, contributes to burnout symptoms such as depersonalization and reduced personal accomplishment, ultimately impairing work performance. In contrast, from the perspective of positive psychology, we integrate conservation of resources theory to propose that digitally hoarded materials serve as potential resources that can enhance future preparedness, support self-development, and buffer against work-related risks—thereby positively influencing job performance (Jia et al. (2025)).

### 5.3 The moderating effects of prevention focus and promotion focus

This study reveals that prevention focus negatively moderates the relationship between workplace digital hoarding and employees’ job performance, whereas the moderating effect of promotion focus is not statistically significant. Previous research has identified multiple motivations underlying employees’ engagement in digital hoarding, including risk mitigation, evidence preservation, information gathering, and self-directed learning (Liu et al., 2023; Sedera et al., 2022; Cheng et al., 2025). Digital hoarding driven by distinct motivations may yield divergent outcomes. Grounded in regulatory focus theory, defensive focus is associated with heightened anxiety in managing risks and may constrain employees’ ability to extract knowledge and support personal development from accumulated digital content. In contrast, promotion focus encourages proactive integration of digital information, thereby fostering greater work vitality and a stronger sense of accomplishment. The empirical findings confirm the negative moderating role of defensive focus, aligning with theoretical predictions. However, the moderating effect of promotion focus was not statistically significant, potentially due to limited statistical power resulting from a smaller sample size. Future studies should validate this effect using larger and more representative samples.

## 6 Theoretical and management implications

### 6.1 Theoretical implications

Based on the theoretical foundations of attentional overload, conservation of resources, and regulatory focus theory, this study investigates the impact, underlying mechanisms, and boundary conditions of digital hoarding in the workplace on work performance. This study employs multiple statistical surveys to demonstrate the positive impact of workplace digital hoarding on employee job performance. This finding aligns with prior literature that acknowledges the beneficial role of digital hoarding and extends existing qualitative insights by providing robust quantitative evidence. In doing so, we reaffirm the conservation of resources theory, which posits that digitally stored resources accumulated through hoarding are functionally meaningful and contribute to enhanced job performance. Second, the study establishes the parallel mediating roles of work burnout and vigor in the relationship between digital hoarding and job performance, thereby bridging theoretical perspectives from cognitive-behavioral frameworks and positive psychology. The empirical results confirm the dual nature of digital hoarding as a double-edged sword, while revealing that its positive effects generally outweigh the negative ones in the workplace context. This advances prior research that has largely relied on single-mediator models, thus fostering greater integration across scholarly domains. Finally, drawing on regulatory focus theory, this study examines how motivational orientations shape the outcomes of digital hoarding and finds that prevention focus significantly attenuates the positive relationship between digital hoarding and job performance. Currently, no quantitative study has tested the boundary role of regulatory focus in this context, making this contribution a novel addition to the literature.

### 6.2 Management implications

The findings of this study provide two practical implications for enterprise media management. First, organizations should actively encourage digital hoarding behavior while guiding employees to effectively process and utilize stored information. While both academic and organizational stakeholders have traditionally expressed concerns that digital hoarding may lead to anxiety, cyberloafing, or compulsive tendencies—thus discouraging such practices—our empirical results offer a more nuanced perspective. When supported by structured interventions, such as regular deletion of redundant files, systematic organization of digital content, and active engagement with saved materials through learning and reflection, digital hoarding can become a functional and beneficial practice. To facilitate this, companies should enhance the bookmarking and archiving features of workplace software and increase the intelligence of digital platforms to improve file management efficiency and promote continuous employee learning. Second, organizations should address defensive forms of digital hoarding by identifying and supporting employees exhibiting maladaptive behaviors. For individuals whose hoarding is driven by risk aversion or high levels of work-related anxiety and burnout, targeted support such as Employee Assistance Programs (EAPs) should be provided. Enterprises may consider appointing specialized psychological counselors focused on digital behavior management to deliver evidence-based interventions, including cognitive-behavioral strategies, to correct dysfunctional patterns and restore psychological wellbeing. Such proactive measures not only mitigate potential downsides but also align digital practices with broader organizational health and productivity goals.

## 7 Limitations and future directions

Although this study yields several robust findings, it is subject to certain limitations that warrant attention in future research. First, the use of a cross-sectional design limits causal inference, as temporal precedence cannot be established, thereby raising concerns about potential reverse causality between workplace digital hoarding and job performance. Future studies should employ longitudinal designs with cross-lagged panel models to better capture the dynamic and reciprocal relationships over time. Second, the sample was drawn exclusively from employees in Chinese enterprises, which may constrain the generalizability of the findings and limit the extent to which theoretical debates can be resolved across diverse cultural contexts. Future research should adopt meta-analytic approaches to synthesize existing evidence and examine cross-cultural variations in the effects of digital hoarding. Finally, the reliance on self-report questionnaires introduces the risk of common method bias and subjective distortions. To enhance measurement validity, future investigations should incorporate objective indicators—such as employees’ actual KPIs and quantifiable metrics of digital storage usage (e.g., file counts or cloud storage volume)—to more accurately assess both job performance and digital hoarding behaviors.

## 8 Conclusion

This study explores the relationship between digital hoarding in the workplace and employee work performance, along with its underlying mechanisms. The key findings are as follows: (1) Workplace digital hoarding positively predicts job performance; (2) Job burnout exerts a negative mediating effect in this relationship, whereas thriving serves as a positive mediator; (3) Prevention focus significantly attenuates the positive association between digital hoarding and job performance, while the moderating role of promotion focus is not statistically significant. These results contribute meaningfully to both theory and practice by advancing our understanding of how digital hoarding interacts with individual emotional states and work outcomes. Furthermore, they offer actionable insights for promoting employee wellbeing through health-oriented media use practices and for enhancing organizational effectiveness via performance-driven media management strategies.

## Data Availability

The raw data supporting the conclusions of this article will be made available by the authors without undue reservation.

## References

[ref1] Agarwal R. Mehrotra A. Pant M. K. Alzeiby E. A. Vishnoi S. K. (2024). Digital photo hoarding in online retail context. An in-depth qualitative investigation of retail consumers. J. Retail. Consum. Serv. 78:103729. doi: 10.1016/j.jretconser.2024.103729

[ref2] Barnes D. C. Collier J. E. (2013). Investigating work engagement in the service environment. J. Serv. Mark. 27, 485–499. doi: 10.1108/JSM-01-2012-0021

[ref3] Çelik C. B. (2025). Who are digital hoarders? Investigation in terms of mental health and personality traits. J. Soc. Serv. Res. 51, 844–856. doi: 10.1080/01488376.2024.2446560

[ref4] Chang C. C. Zhuang W. L. Hung C. W. Huan T. C. (2024). Investigating the influence of thriving at work on hotel employees’ service performance with the moderating effect of leader-member exchange. Int. J. Hosp. Manag. 119:103736. doi: 10.1016/j.ijhm.2024.103736

[ref5] Cheng S. Q. Li J. J. Xie Y. (2025). Saving but not acting: how hoarding health information on social media inhibits real-world health behavior? Int. J. Hum. Comput. Interact. doi: 10.1080/10447318.2025.2575101

[ref6] Demerouti E. Verbeke W. J. M. I. Bakker A. B. (2005). Exploring the relationship between a multidimensional and multifaceted burnout concept and self-rated performance. J. Manage. 31, 186–209. doi: 10.1177/0149206304271602

[ref7] Duan W. Fei Y. Zhao J. Guo X. (2018). Incremental validity of the comprehensive inventory of thriving in predicting self-reporting mental and physical health among community populations. J. Health Psychol. 25, 1366–1373. doi: 10.1177/1359105318755265, 29402146

[ref8] Duan W. Guan Y. Gan F. (2016). Brief inventory of thriving: a comprehensive measurement of wellbeing. Chin. Sociol. Dialogue 1. doi: 10.1177/2397200916665230

[ref9] Hart R. (2024). Prosocial behaviors at work: key concepts, measures, interventions, antecedents, and outcomes. Behav. Sci. 14:78. doi: 10.3390/bs14010078, 38275361 PMC10813621

[ref10] Head J. Helton W. S. (2014). Sustained attention failures are primarily due to sustained cognitive load not task monotony. Acta Psychol. 153, 87–94. doi: 10.1016/j.actpsy.2014.09.007, 25310454

[ref11] Higgins E. T. (1997). Beyond pleasure and pain. Am. Psychol. 52, 1280–1300. doi: 10.1037//0003-066x.52.12.1280, 9414606

[ref12] Jia M. X. Zhao Y. C. Zhang X. Y. Wu D. W. (2025). “That looks like something I would do”: understanding humanities researchers’ digital hoarding behaviors in digital scholarship. J. Doc. 81, 24–55. doi: 10.1108/JD-01-2024-0004

[ref13] Karagöz D. Ramkissoon H. (2024). Loneliness, travel nostalgia, subjective well-being and prevention regulatory focus: a moderated mediation model analysis. Curr. Issue Tour. 27, 217–233. doi: 10.1080/13683500.2023.2175201

[ref14] Koklic M. K. Kukar-Kinney M. Vida I. (2025). Dark sides of digital asset consumption and consumer well-being: impact of psychological ownership. J. Consum. Behav. 24, 2351–2371. doi: 10.1002/cb.70011

[ref15] Liu Y. Chi X. L. Xin X. M. (2023). Storing, not reading: investigating the link between upward social comparison via social media and digital hoarding behavior in Chinese youth. Psychol. Res. Behav. Manag. 16, 5209–5224. doi: 10.2147/PRBM.S441859, 38152591 PMC10752025

[ref9002] Liu Y. Liu Y. R. (2025). Hoarding knowledge or hoarding stress? Investigating the link between digital and cognitive failures among Chinese college students. Front Psychol, 15:1518860. 39949973 10.3389/fpsyg.2024.1518860 PMC11821920

[ref16] Liu Y. Liu Y. L. Feng Y. P. (2024). From clicks to calm: investigating the link between mindfulness and digital hoarding behavior among Chinese youth. Psychol. Res. Behav. Manag. 17, 3283–3297. doi: 10.2147/PRBM.S473523 39346091 PMC11438452

[ref17] Luxon A. M. Hamilton C. E. Bates S. Chasson G. S. (2019). Pinning our possessions: associations between digital hoarding and symptoms of hoarding disorder. J. Obsessive Compulsive Related Disord. 21, 60–68. doi: 10.1016/j.jocrd.2018.12.007

[ref18] Maduku D. K. (2024). How environmental concerns influence consumers’ anticipated emotions towards sustainable consumption: the moderating role of regulatory focus. J. Retail. Consum. Serv. 76:103593. doi: 10.1016/j.jretconser.2023.103593

[ref9001] Maslach C. (2012). Burnout in the workplace: a global problem in need of solution. Int. J. Psychol. 47:549.

[ref19] McKellar K. Sillence E. Neave N. Briggs P. (2020). There is more than one type of hoarder: collecting, managing and hoarding digital data in the workplace. Interact. Comput. 32, 209–220. doi: 10.1093/iwc/iwaa015

[ref20] McKellar K. Sillence E. Neave N. Briggs P. (2024). Digital accumulation behaviours and information management in the workplace: exploring the tensions between digital data hoarding, organizational culture and policy. Behav. Inform. Technol. 43, 1206–1218. doi: 10.1080/0144929X.2023.2205970

[ref21] Mehrotra A. Basahel S. Virmani N. Briamonte M. F. (2025). Beyond storage: a TCV-SECI framework for digital photo hoarding and knowledge transformation. J. Knowl. Manag. 29, 2084–2103. doi: 10.1108/JKM-10-2024-1230

[ref22] Miao L. Gazi M. A. Karima B. (2025). The job performance and job burnout relationship: a panel data comparison of four groups of academics’ job performance. Front. Public Health 12:1460724. doi: 10.3389/fpubh.2024.1460724 39830187 PMC11738915

[ref23] Mu W. L. Cui S. Y. Deng F. F. Liu T. Y. (2025). Validity and reliability of the Chinese version of digital hoarding scale. Res. Soc. Work. Pract. 35, 724–733. doi: 10.1177/10497315241267202

[ref24] Neave N. Briggs P. McKellar K. Sillence E. (2019). Digital hoarding behaviours: measurement and evaluation. Comput. Human Behav. 96, 72–77. doi: 10.1016/j.chb.2019.01.037

[ref25] Podsakoff P. M. Ahearne M. Mackenzie S. B. (1997). Organizational citizenship behavior and the quantity and quality of work group performance. J. Appl. Psychol. 82, 262–270. doi: 10.1037/0021-9010.82.2.262, 9109284

[ref26] Porath C. Spreitzer G. Gibson C. Garnett F. G. (2012). Thriving at work: toward its measurement, construct validation, and theoretical refinement. J. Organ. Behav. 33, 250–275. doi: 10.1002/job.756

[ref27] Prentice C. Thaichon P. (2019). Revisiting the job performance – burnout relationship. J. Hosp. Mark. Manag. 28, 807–832. doi: 10.1080/19368623.2019.1568340

[ref28] Ryan R. M. Deci E. L. (2001). On happiness and human potentials: a review of research on hedonic and Eudaimonic well-being. Ann. Rev. Psychol. 52, 141–166. doi: 10.1146/ANNUREV.PSYCH.52.1.141, 11148302

[ref29] Sedera D. Lokuge S. Grover V. (2022). Modern-day hoarding: a model for understanding and measuring digital hoarding. Inf. Manag. 59:103700. doi: 10.1016/j.im.2022.103700

[ref30] Sillence E. Dawson J. A. Brown R. D. McKellar K. Neave N. (2023). Digital hoarding and personal use digital data. Hum. Comput. Interact. 38, 1–20. doi: 10.1080/07370024.2023.2293001, 41307611

[ref31] Su R. Tay L. Diener E. (2014). The development and validation of the comprehensive inventory of thriving (CIT) and the brief inventory of thriving (BIT). Appl. Psychol. Health Wellbeing 6, 251–279. doi: 10.1111/aphw.12027, 24919454

[ref32] Sweeten G. Sillence E. Neave N. (2018). Digital hoarding behaviours: underlying motivations and potential negative consequences. Comput. Human Behav. 85, 54–60. doi: 10.1016/j.chb.2018.03.031

[ref33] Tandoc E. C. Jr. Lou C. Min V. L. H. (2019). Platform-swinging in a poly-social-media context: how and why users navigate multiple social media platforms. J. Comput.-Mediat. Commun. 24, 21–35. doi: 10.1093/jcmc/zmy022

[ref34] Tarsuslu S. (2024). Does corporate governance affect thriving at work? Employees’ roles in knowledge sharing and prosocial motivation. Anadolu Üniversitesi Sosyal Bilimler Dergisi 24, 1475–1498. doi: 10.18037/ausbd.1462758

[ref35] Thorpe S. Bolster A. Neave N. (2019). Exploring aspects of the cognitive behavioural model of physical hoarding in relation to digital hoarding behaviours. Digit. Health 5. doi: 10.1177/2055207619882172, 31636918 PMC6785915

[ref36] Tugtekin U. Tugtekin E. B. (2025). Influential factors on academics’ digital hoarding behaviours: an exploratory PLS-SEM research. Behav. Inf. Technol. 44, 1667–1680. doi: 10.1080/0144929X.2024.2368701

[ref37] Van Bennekom M. J. Blom R. M. Vulink N. Denys D. (2015). A case of digital hoarding. BMJ Case Rep. 2015:bcr2015210814. doi: 10.1136/bcr-2015-210814, 26452411 PMC4600778

[ref38] Van der Vegt G. S. Janssen O. (2003). Joint impact of interdependence and group diversity on innovation. J. Manage. 29, 729–751. doi: 10.1016/S0149-2063(03)00033-3

[ref39] Vinoi N. Shankar A. Mehrotra A. Kumar J. Azad N. (2024). Enablers and 25 rganizati of digital hoarding behaviour. An application of dual-factor theory and regret theory. J. Retail. Consum. Serv. 77:103645. doi: 10.1016/j.jretconser.2023.103645

[ref40] Vital F. Janzen I. McGrenere J. (2018). “Hoarding and minimalism: tendencies in digital data preservation” in Proceedings of the 2018 CHI conference on human factors in computing systems. Montreal, Canada: Assoc Comp Machinery; ACM SIGCHI.

[ref41] Wang H. X. Miao P. Jia H. Y. Lai K. S. (2023). The dark side of upward social comparison for social media users: an investigation of fear of missing out and digital hoarding behavior. Soc. Media Soc. 9. doi: 10.1177/20563051221150420

[ref42] Waterman A. S. (2008). Reconsidering happiness: a eudaimonist’s perspective. J. Posit. Psychol. 3, 234–252. doi: 10.1080/17439760802303002

[ref43] Williams L. J. Anderson S. E. (1991). Job satisfaction and organizational commitment as predictors of organizational citizenship and in-role behaviors. J. Manage. 17, 601–617. doi: 10.1177/014920639101700305

[ref44] Wu D. W. Zhao Y. C. Wang X. L. Song S. J. Lian J. W. (2024). Digital hoarding in everyday hedonic social media use: the roles of fear of missing out (FoMO) and social media affordances. Int. J. Hum. Comput. Interact. 40, 5399–5414. doi: 10.1080/10447318.2023.2233139

[ref45] Zaremohzzabieh Z. Abdullah H. Ahrari S. Abdullah R. Nor S. M. M. (2024). Exploration of vulnerability factors of digital hoarding behavior among university students and the moderating role of maladaptive perfectionism. Digit. Health 10. doi: 10.1177/20552076241226962, 38298527 PMC10829496

