# Supplementary material for: Exploring the effect of digital hoarding in the workplace on employee work performance
Source: Front Psychol. 2025 Dec 18;16:1198825. doi: 10.3389/fpsyg.2025.1198825 (PMC12756427; doi:10.3389/fpsyg.2025.1198825)
Supplement: Supplementary file 1 [file Table_1.docx]

**Appendix:** **Instruments and items used**

The items of the main variables are presented in Supplementary Table 1, with the digital hoarding scales used in the main study and the robustness test labeled as DHW1 and DHW2, respectively.

Supplementary Table 1. Instruments and items used

| Variable/Source | Item | Variable/Source | Item |
| --- | --- | --- | --- |
| WP  (Williams, L. J. & Anderson, S. E., 1991, Podsakoff, P. M., Ahearne, M. & Mackenzie, S. B., 1997 and Van der Vegt, G. S. & Janssen, O., 2003) | I can competently fulfill the duties assigned to me. | DHW1  (Neave, N. et al, 2019) | I will try to expand the storage space of the device or application to increase the number of files. |
|  | I can carry out the responsibilities clearly stated in the job description. |  | I will try to use a variety of media management methods to increase the number of files. |
|  | I complete the tasks expected of me. |  | I will add content that I am interested in or find useful to the device or application. |
|  | I meet the formal performance requirements of the job. |  | I will keep some videos, articles, or records for a long time because they are interesting. |
|  | I fulfill the obligatory aspects of the work. |  | I will keep some videos, articles, or records because of their potential use. |
|  | I can keep up with the task schedule as required by the project timeline. |  | I will not delete files from the device even after a long time has passed. |
|  | I am able to ensure the quality of task completion. | DHW2  (Sillence E. et al., 2023) | I will store files that others might not keep. |
|  | I won't bring excessive costs during the process of completing the task. |  | Even if they are not related to current needs, I tend to save some files. |
|  | I exhibit a high level of responsibility throughout the task completion process. |  | Storing files gives me a sense of security. |
|  | I can maintain a high level of enthusiasm for work tasks. |  | I struggle to organize the files I collect. |
|  | I can offer new ideas for improving technology, processes, services or production or sales. |  | By deleting some files, I might lose something. |
|  | I can promptly identify problems or challenges and address unresolved issues. |  | Due to work requirements, we may not be able to delete certain files. |
|  | I can come up with original and feasible solutions to problems. | TV  (Porath et al., 2012) | I feel energetic. |
|  | I can strive to gain the approval of my new ideas from other colleagues. |  | I feel so energetic that I want to burst. |
|  | I can strive to get my new ideas or plans approved by my superiors. |  | I am full of energy and vigor. |
|  | I can actively support others' new ideas or plans. |  | I look forward to every day. |
|  | I can get my new ideas or plans for helping others supported by my colleagues or superiors. |  | I feel full of energy. |
|  | I can proactively assess the advantages and disadvantages of my new ideas or plans. |  | I feel sharp and alert. |
|  | I can proactively assess others' new ideas and offer suggestions. |  | I don't feel energetic. (reverse score) |
|  | I can actively promote the implementation of my new ideas or plans in my work. |  | I feel my energy is exhausted. (reverse score) |
|  | I can proactively promote the implementation of others' new ideas or plans in the workplace. |  | I feel sleepy. (reverse score) |
|  | I can adopt new technology to reduce costs and improve efficiency. |  | I lack energy. (reverse score) |
|  | I can plan and allocate work content reasonably, saving working time. |  | I am experiencing a rapid growth process. |
|  | I can improve the service methods based on the job requirements of the clients. |  | I am growing positively. |
|  | When problems arise in innovation, I can manage to solve them as soon as possible. |  | I haven't grown much recently. (reverse score) |
|  | I can effectively handle emergencies that I have never encountered before. |  | I am stagnant. (reverse score) |
|  | I can independently summarize feasible new working methods. |  | I'm glad to witness the slightest progress of my thoughts. |
|  | I can identify unique and feasible techniques. |  | As time goes by, I keep learning and improving. |
|  | I can solve difficult problems creatively or my innovations have been patented. |  | I have found a new path for growth. |
|  | I help my colleagues deal with their life problems. |  | I no longer study. (Reverse score) |
|  | I can provide information that is beneficial to others. |  | I'm becoming more and more mature. |
|  | I can encourage others to overcome difficulties. |  | I'm no longer making progress. (Reverse score) |
|  | I have a positive attitude towards the organization. |  | I often study by myself. |
|  | I am loyal to the organization. |  | I see myself constantly improving. |
|  | I have devoted a considerable amount of energy to my studies. |  | I think I'm continuously developing. |
|  | I attach more importance to the quality of learning. |  | I'm not making progress. (Reverse score) |
|  | I maintain a high learning efficiency. | PRE-F  (Higgins E. T., 1997) | I focus on preventing negative events from happening in my life. |
|  | I strive to broaden my knowledge base. |  | I'm worried that I won't be able to fulfill all my responsibilities and obligations. |
|  | I have a strong ability to carry out learning tasks. |  | I often think about the person I'm afraid I might become in the future. |
| JB  (Maslach C., 1986 and Maslach C. & Jackson S.E., 1981) | I'm extremely tired. |  | I often worry that I won't be able to achieve my goals. |
|  | I don't care about the inner feelings of my work partners. |  | I often think about how I can avoid failure in life. |
|  | I can effectively solve the problems of my work partners (reverse score). |  | I tend to avoid losses rather than seek gains. |
|  | I'm worried that work will affect my mood. |  | I often imagine myself going through those bad things that I'm afraid might happen to me. |
|  | My work partners often complain about me. |  | At present, my main goal is to avoid failure in my career. |
|  | I often feel exhausted. |  | I see myself as someone striving to fulfill my duties and obligations. |
|  | I work with a cynical attitude. I can create a relaxed and lively working atmosphere (reverse score). | PRO-F  (Higgins E. T., 1997) | I often imagine how I will realize my hopes and ambitions. |
|  | At the end of a day's work, I feel extremely fatigued. |  | I often think of the person I hope to become in the future. |
|  | I often scold my work partners. |  | My attention is usually focused on the success I hope to achieve in the future. |
|  | I'm very excited after solving the problems of my work partners (reverse score). |  | I often think about how I will achieve success in my career. |
|  | Recently, I've been a little depressed. |  | My main goal in my studies at present is to realize my career aspirations. |
|  | I often refuse the requests of my work partners. |  | I consider myself a person who strives to reach my ideal self. |
|  | I've accomplished many meaningful work tasks (reverse score). |  | Generally speaking, I focus on achieving positive results in life. |
|  | I can effectively influence others through my work (reverse score). |  | I often imagine myself experiencing the good things I hope will happen to me. |
|  | - |  | In general, I am more inclined to achieve success than to avoid failure. |
